# Community Health workers United to Reduce Colorectal cancer and cardiovascular disease among people at Higher risk (CHURCH): study protocol for a randomized controlled trial

**DOI:** 10.1186/s13063-024-08110-z

**Published:** 2024-04-26

**Authors:** Olajide Williams, Tina Ting, Lisa Matthews, Gladys Block, Torin Block, Jeanne Teresi, Joseph Eimicke, Jian Kong, Stephanie Silver, Joseph Ravenell, Janhavi Mallaiah, Soujanya Jammalamadaka, Laura Maudene Nelson, Wahida Karmally, Sidney Hankerson

**Affiliations:** 1 Columbia University Irving Medical Center, 710 West 168th Street, New York, NY 10032 USA; 2 NutritionQuest and Turnaround Health, Berkeley, CA USA; 3 Division of Medicine, Data Coordinating Center Unit, Columbia University Irving Medical Center, Stroud Center at New York State Psychiatric Institute, 622 West 168th Street, New York, NY 10032 USA; 4 New York University Grossman School of Medicine, Manhattan, NY USA; 5 Icahn School of Medicine at Mount Sinai, Manhattan, NY USA

**Keywords:** Colorectal cancer, Community-based research, Health disparities, Randomized trial, Community health workers, Inflammation, Dietary pattern

## Abstract

**Background:**

Colorectal cancer (CRC) is the second most lethal cancer in the United States (U.S.) with the highest incidence and mortality rates among African Americans (AAs) compared to other racial groups. Despite these disparities, AAs are the least likely to undergo CRC screening, have precancerous colorectal polyps removed, and have CRC detected at stages early enough for curative excision. In addition, compelling evidence links inflammatory dietary patterns to increased CRC and cardiovascular disease risk. Studies show that AA churches can successfully engage in health promotion activities including those related to cancer control. The current study seeks to leverage church-placed Community Health Workers (CHWs) to increase CRC screening and reduce CRC risk.

**Design and methods:**

We aim to (1) increase guideline concordant CRC screening uptake using church-placed CHWs trained in screening with a validated instrument, Brief Intervention using Motivational Interviewing, and Referral to Treatment (SBIRT); and (2) reduce dietary risk factors (inflammatory dietary patterns) linked to CRC. The latter will be addressed by culturally adapting an existing, web-based lifestyle program called Alive!. Using a Hybrid Type 1 Implementation-Effectiveness cluster randomized design, we will randomize 22 AA churches into either the dual intervention arm (CHW-led SBIRT intervention plus Alive!) or a usual care arm comprised of CRC prevention educational pamphlets and a list of CRC screening sites. We will recruit 440 subjects and evaluate the effects of both arms on screening uptake (colonoscopy, fecal DNA) (primary outcome) and dietary inflammation score (secondary outcome) at 6-month follow-up, and Life Simple7 (LS7)—a cardiovascular disease (CVD) risk score—at 6 months and 1 year (secondary outcome). Finally, guided by a racism-conscious adaptation of the Consolidated Framework for Implementation Research (CFIR), we will conduct a mixed-methods process evaluation with key stakeholders to understand multi-level influences on CRC screening and CVD risk behaviors.

**Discussion:**

Church-placed CHWs are trusted influential connectors between communities and health systems. Studies have shown that these CHWs can successfully implement health prevention protocols in churches, including those related to cancer control, making them potentially important community mediators of CRC screening uptake and CRC/CVD risk reduction.

**Trial registration:**

NCT05174286; clinicaltrials.gov; August 31^st^, 2023

## Administrative information

**Table:** 

Title {1}	Community Health workers United to Reduce Colorectal cancer and cardiovascular disease among people at Higher risk (CHURCH): study protocol for a randomized controlled trial
Trial registration {2a and 2b}	NCT05174286; clinicaltrials.gov; August 31st, 2023
Protocol version {3}	IRB-AAAT9307; May 2, 2023
Funding {4}	This study is funded by the National Institute on Minority Health and Health Disparities/NIH/DHHS (1P50MD017341-01)
Author details {5a}	**Olajide Williams, MD, MS** Ow11@cumc.columbia.edu Columbia University Irving Medical Center 710 West 168th Street New York, NY 10032 **Tina Ting, MPH (Corresponding Author)** Tyt2108@cumc.columbia.edu Columbia University Irving Medical Center 710 West 168th Street New York, NY 10032 **Lisa Matthews, MPH, MS, Ed.D** Lam2160@cumc.columbia.edu Columbia University Irving Medical Center 710 West 168th Street New York, NY 10032 **Gladys Block, PhD** Gblock@turnaroundhealth.com NutritionQuest and Turnaround Health Berkeley, CA, USA **Torin Block** Tblock@nutritionquest.com NutritionQuest and Turnaround Health Berkeley, CA, USA **Jeanne Teresi, Ed.D, PhD** Jat61@cumc.columbia.edu Columbia University Irving Medical Center Division of Medicine, Data Coordinating Center Unit Stroud Center at New York State Psychiatric Institute 622 West 168th Street New York, NY 10032 **Joseph Eimicke, MS** Jpe2114@cumc.columbia.edu Columbia University Irving Medical Center Division of Medicine, Data Coordinating Center Unit 622 West 168th Street New York, NY 10032 **Jian Kong, MS** Jk71@cumc.columbia.edu Columbia University Irving Medical Center Division of Medicine, Data Coordinating Center Unit 622 West 168th Street New York, NY 10032 **Stephanie Silver, MPH** Sal2244@cumc.columbia.edu Columbia University Irving Medical Center Division of Medicine, Data Coordinating Center Unit 622 West 168th Street New York, NY 10032 **Joseph Ravenell, MD, MS** Joseph.ravenell@nyulangone.org New York University Grossman School of Medicine, New York **Janhavi Mallaiah, MD, MPH, MS, Ed.D** Jm4498@cumc.columbia.edu Columbia University Irving Medical Center 710 West 168th Street New York, NY 10032 **Soujanya Jammalamadaka** Sj3161@cumc.columbia.edu Columbia University Irving Medical Center 710 West 168th Street New York, NY 10032 **Laura Maudene Nelson** Mln2@cumc.columbia.edu Columbia University Irving Medical Center 710 West 168th Street New York, NY 10032 **Wahida Karmally, Dr.PH, MS, RDN, CDCES, CLS, FNLA** Wk2@cumc.columbia.edu Columbia University Irving Medical Center 710 West 168th Street New York, NY 10032 **Sidney Hankerson, MD, MBA** Sidney.hankerson@mountsinai.org Icahn School of Medicine at Mount Sinai, New York
Name and contact information for the trial sponsor {5b}	Sponsor: National Institute on Minority Health and Health Disparities: Award Number: P50MD017341 Multiple Principal Investigators (MPIs) Elizabeth Gross Cohn, PHD Dawn Hershman, MD Erica Phillips, MD Daichi Shimbo, MD Mary Beth Terry (contact), PHD Olajide Williams, MD (and CHURCH Trial PI)
Role of sponsor {5c}	Study sponsor did not participate in study design, collection, management, analysis, and interpretation of data, writing of the report, or the decision to submit the report for publication. Study sponsor will also not have ultimate authority over the aforementioned activities

## Introduction

### Background and Rationale {6a}

African American (AA) adults are more likely to contract and die from colorectal cancer (CRC) than any other racial group in the United States (U.S). Despite data showing that screening colonoscopies are associated with strong reductions in CRC incidence and mortality, [1, 2] AA are the least likely to be screened [3], or adhere to risk reducing programs for CRC. There is a dearth of sustainable multi-level interventions targeting AAs that include a dual focus on reducing the burden of CRC and its cardiovascular disease (CVD) risk factors—two devastating diseases with a common social context and similar health disparity profiles. Given the influence and reach of churches and the mediating role of community health workers (CHWs) between churches, communities, and health systems, we propose to test a cluster randomized church-placed intervention, at the center of which are CHW-congregant dyads, to increase guideline concordant CRC screening and decrease related lifestyle risk factors, while understanding contextual factors influencing our outcomes of interest.

#### Burden of colorectal cancer among African Americans

CRC is the second most common cause of cancer deaths in the U.S and a contributor to premature mortality [4]. AAs have the highest incidence and mortality from CRC and the highest likelihood of being diagnosed at an earlier age and with more advanced disease [5] compared to other race-ethnic groups [5]. In fact, late-stage CRC accounts for more than 60% of the disparity in mortality [6]. The unexpected death of Chadwick Boseman—lead actor of the Marvel movie “Black Panther”—at the age of 43 years from CRC highlighted these statistics, exposing a lack of awareness about the disease.

According to the New York City Department of Health Mental Hygiene Bureau of Vital Statistics, neighborhood has a dramatic effect on CRC incidence rates, with a 30% higher age-adjusted incidence rate among blacks living in the poorest neighborhoods. Indeed, racial residential segregation has been shown to impact CRC outcomes, and AA patients living in more segregated counties are more likely to present with advanced disease, have lower likelihood of surgical resection, and have worse cancer-specific survival [7].

#### Racial disparities in colorectal cancer screening

The disparities discussed in the previous section are not related to genetic differences, but to structural inequities in access to CRC screening, utilization of CRC screening, and quality of CRC treatment [8, 9]. In fact, the lower screening rates by any method among AAs compared to Whites is the major modifiable driver of CRC disparities [10]. Moreover, these lower screening rates persist in equal access settings and among insured subjects, making barriers related to healthcare mistrust, health literacy, and perceived risk important to address. For example, although 25% of patients with CRC have a family history, AAs are less likely to know their paternal history or disclose findings of colon polyps to their relatives compared to Whites [11], contributing to inequity in screening.

#### Strategies to improve colorectal cancer screening uptake among African Americans

Physician recommendations, physician-directed multimodal education with reminders, increased health insurance coverage, the use of patient navigators, and tailored patient education have shown modest benefits on CRC screening uptake [12]. Notwithstanding, there is a scarcity of rigorously tested approaches leveraging multiple socio-ecological levels of influence (individual, relationship, institutional, community, societal) at the same time, which increases the likelihood of a greater and more sustained impact [13] and broadens the lens through which the intervention can anticipate pitfalls. For example, one intervention which focused on the institutional level by targeting greater availability of colonoscopists inadvertently increased colonoscopy rates among Whites and decreased rates among AAs [14]. Other studies suggest that offering stool-based tests such as the fecal immunochemical test (FIT) may improve screening rates [15] suggesting that a greater emphasis on shared decision-making regarding guideline screening options is beneficial. For individuals aged 45–75 years, these options include (1) high-sensitivity guaiac fecal occult blood test (gFOBT) or FIT every year; (2) stool DNA test (sDNA)-FIT every 1 to 3 years; (3) computed tomography (CT) colonography every 5 years; (4) flexible sigmoidoscopy every 5 years; (5) flexible sigmoidoscopy every 10 years + FIT every year; and (6) colonoscopy screening every 10 years.

### Objectives {7}

The overarching goal of this proposal is to create a scalable community-academic partnership model for CRC and CVD prevention for AA communities. Our dual intervention approach allows us to target two major killers of AAs. We aim to (1) increase guideline concordant CRC screening uptake using a CHW-led Screening, Brief Intervention, and Referral to Treatment (SBIRT) intervention (primary outcome); and (2) reduce dietary and CVD risk factors linked to CRC (secondary outcomes). The latter will be addressed by culturally adapting an existing, evidence-based, web-based lifestyle program called Alive!, which has been shown in several RCTs to improve CVD outcomes [16]. Using a Hybrid Type 1 Implementation-Effectiveness design, we will randomize 22 AA churches (440 subjects) into a cluster RCT to accomplish the following:

#### Aim 1: To compare the effect of SBIRT (intervention) to referral as usual (RAU) (usual care) on guideline-concordant CRC screening uptake

We hypothesize that the intervention will lead to increased CRC screening uptake (colonoscopy, fecal DNA) (primary outcome) compared to RAU at 6 months.

#### Aim 2: To evaluate the effect of a culturally adapted Alive! program (CAP) incorporated into the intervention arm on dietary inflammation score (DIS)

Participants in the intervention group will participate in CAP following the completion of the SBIRT intervention. We hypothesize that participants in the CAP arm will have lower DIS scores compared to RAU at 6 months.

#### Aim 3: To evaluate the effect of CAP on changes in Life Simple-7 (LS7) scores

We hypothesize that participants in the CAP arm will have improvement in LS7 scores compared to RAU at 6 months and 1 year.

#### Aim 4: To examine the multi-level contextual mechanisms and factors influencing CHW effectiveness, reach, and implementation of CRC screening uptake and CAP activities

Guided by a racism-conscious adaptation of the Consolidated Framework for Implementation Research (CFIR) [17, 18], we will conduct a mixed methods process evaluation with key stakeholders to understand multi-level influences on CRC screening and CVD risk behaviors. This information will inform future dissemination and scale-up of this intervention.

### Trial design {8}

**Fig. 1:**
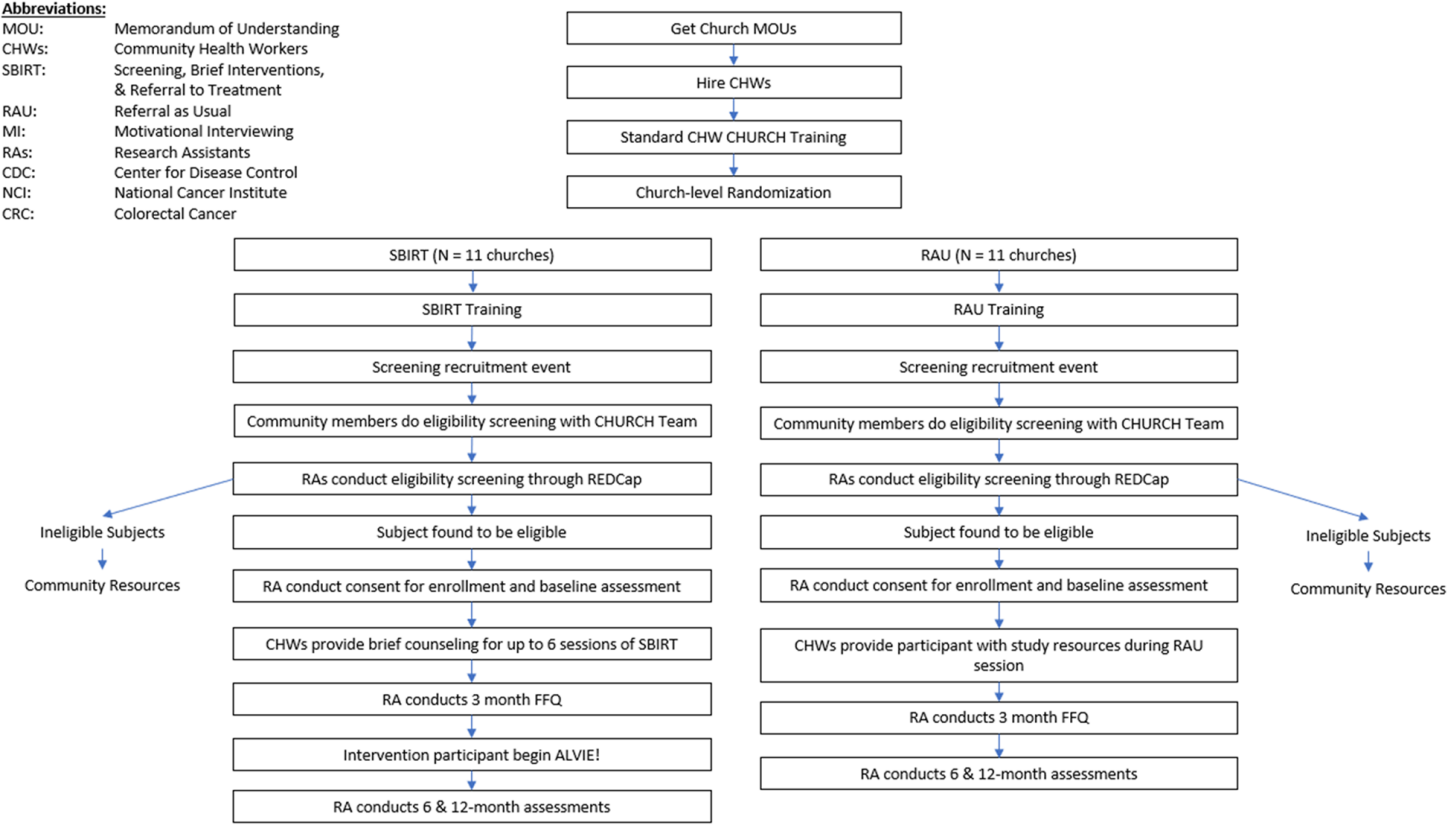
Trial design overview

We will use a hybrid type 1 effectiveness-implementation design [19] and a community-based participatory research approach (CBPR) [20] to engage AA churches and study participants. We will conduct a two-arm, cluster RCT in 22 AA churches comparing the effectiveness of CHW-delivered SBIRT (*n* = 11 churches) to RAU (*n* = 11 churches) on CRC screening uptake (colonoscopy, stool-based test) (primary outcome) (Fig. 1). We will also examine the effect of an evidence-based CAP program incorporated into the intervention arm on DIS and on changes to CVD risk captured by LS7 scores (secondary outcomes). Lastly, we will utilize a concurrent, mixed-methods approach to assess cultural and contextual factors including barriers and facilitators of screening uptake and implementation in churches. The CFIR [21] will guide our implementation and process evaluation. We will utilize an adapted version of CFIR, which incorporates a race-conscious lens [17, 22] to generate semi-structured interviews with key stakeholders.

There are 3 waves of data collection: baseline, 6 months, and 12 months; for the CAP, there will be an additional 3-month baseline collection of the FFQ.

## Methods: participants, interventions, and outcomes

### Study setting {9}

This study will be conducted in 22 AA churches throughout New York City. Churches with at least 500 active members where CHWs currently attend will be eligible. We will create an eligibility score based on estimates of how many in the church congregation are 45 years and older. Other criteria will include having an African American congregation with greater than two thirds who identify as Black. We have received letters of support from 25 churches which includes 3 buffer churches.

### Eligibility criteria {10}

#### General eligibility for participation in RCT

General eligibility for study participation includes as follows: (1) English-speaking; (2) self-identifying as Black; (3) Aged 45 years to 75; (4) not up to date with CRC screening; (5) working telephone; (6) can provide informed consent.

#### Eligibility for CRC screening

Based on USPSTF guidelines, eligibility for CRC screening include the following: Church members not compliant with the following screening recommendations beginning at age 45 and up to age 75 are eligible: (1) high-sensitivity gFOBT or FIT every year; (2) sDNA-FIT every 1 to 3 years; (3) CT colonography every 5 years; (4) flexible sigmoidoscopy every 5 years; (5) flexible sigmoidoscopy every 10 years + FIT every year; (6) colonoscopy screening every 10 years.

#### Exclusion criteria

Screening questions include a family history of CRC or colorectal polyps. Participants who indicate they have an immediate family member who has or had colorectal cancer (i.e., father, mother, brother, or sister) will be excluded from participation in the study. These individuals will be directly referred for screening at one of the study’s partner hospitals.

### Informed consent {26a}

Upon completing the eligibility screener, a scoring algorithm programmed into REDCap will determine the participant’s eligibility for participation in the study. The research assistant administering the screener will then verify the participant’s eligibility scoring. Informed consent will be administered to eligible participants by trained research assistants (RAs) using REDCap. Informed consent will be obtained either in person directly following the screener at church lead recruitment events or prior to the scheduled baseline assessment, depending on the availability of the participant. This informed consent process will begin with a concise presentation of the key information about the research study, including a description of the study purpose and components, potential risks, and benefits of participating. During this process, potential subjects will have an opportunity to discuss the information provided and will be informed of their right to refuse to answer questions in the assessments or withdraw from the study if they choose to, without affecting their access to health services.

### Additional consent provisions for collection and use of participant data and biological specimens {26b}

A description of collection of biological specimens during point of care tests is included in the informed consent form.

## Interventions

### Explanation for the choice of comparators {6b}

This study will compare the effect of SBIRT and CAP (Intervention) to RAU (usual care) on guideline-concordant CRC screening uptake. SBIRT is an evidence-based approach, originally designed for people at risk of developing mental disorders, [23] comprised of three components: (1) Screening with a validated instrument, (2) Brief Intervention, (3) Referral to Treatment. Alive! is an automated platform that includes step-by-step individualized tailoring, feedback, and weekly guidance through interactive emails focused on increasing physical activity, and improving eating patterns. In contrast, the RAU group will receive the most common form of referral from church-based screening events, which includes educational brochures, community resources, and a list of CRC screening sites.

### Intervention description {11a}

#### Screening, brief intervention, and referral to treatment

Multiple studies have found SBIRT to be effective in increasing referral rates for mental health-related treatment, and adherence to follow-up [24–26]. Given the multi-generation trauma related to self-reported discrimination and resulting distrust of the medical system among AA populations, [27] we decided to adapt SBIRT for CRC screening because it embraces Trauma-Informed Care (TIC).

The screening component will include items that will be adapted from the Behavioral Risk Factor and Surveillance System CRC screening tool to screen guideline adherence at baseline and 6 months.

The brief intervention component of SBIRT will be Motivational Interviewing (MI), which has been shown to be effective at improving health outcomes among AAs in church-based settings when delivered by paraprofessionals [28]. MI is an empirically tested, person-centered, behavior change intervention designed to guide, elicit, and strengthen motivation for change [29]. It decreases ambivalence and increases motivation for treatment. Studies of MI have found (1) an increase in CRC screening; (2) an increase in healthy eating patterns; and (3) improved CVD risk factor control among AA adults [21, 30, 31].

CHWs will provide up to six MI sessions over a 3-month period. The exact number of sessions will vary based on participant needs. The sessions will occur in person or virtually via phone/zoom. Data will be collected on method of delivery. MI sessions will help eligible participants move through the various stages of change associated with seeking screening for CRC. In these sessions, CHWs will establish rapport through open-ended questions, Affirmations, Reflections, and Summary statements (OARS), review reasons for non-compliance with guideline screening, and assess motivation and confidence regarding obtaining a screening test and elicit cognitive, emotional, and socio-economic-related barriers and facilitators of screening. CHWs will also work with participants to consider the “pros” and “cons” of screening, provide screening options for the participant based on the nature of barriers elicited from them and addressing social determinants of health (SDoH)-related needs, assess participant’s values and goals and help them link their current predisposition to screening to their goals, and summarize what was discussed in order to clarify an action plan.

The final component of SBIRT involves actual referrals for CRC screening. This begins with a determination of the individual’s health insurance status. Persons without insurance will be enrolled with the assistance of CHWs (who are certified New York State Insurance Navigators) into New York State health plans (insurance exchange or Medicaid). Individuals who are ineligible for health insurance will be referred to our network of local clinics providing CRC screening services regardless of the ability to pay. CHWs will secure a gastrointestinal (GI) clinic appointment for subjects who are guideline discordant for CRC screening within 4 weeks of the assessment (average appointment lag time within our referral networks). CHWs will refer subjects to our network of GI clinics based on subject preference and insurance status. They will also place a reminder call on the day before the appointment. We will be partnering with Harlem Hospital to screen participants for CRC.

#### Culturally adapted Alive! program (CAP)

Guided by the Ecological Validity Model, we will culturally adapt the Alive! Program—a cost-effective, lifestyle coaching web-based platform [16, 32–34] incorporating the transtheoretical model. Alive! is listed on the National Cancer Institute (NCI) Evidence-Based Cancer Control Program (EBCCP) with high RE-AIM (Reach, Effectiveness, Adoption, Implementation, Maintenance) scores for real-world translation. The current version of Alive! is delivered via an individualized website on a desktop or mobile platform and contains built-in assessments of baseline diet (using a modified Food Frequency Questionnaire, FFQ) [35] and physical activity (using an adapted Block Physical Activity Questionnaire) [36] for personalized feedback. We will adapt the fully automated program from its current 36 weeks to 12 weeks (we considered 3-month attrition rates) [37] and enhance the intensity of dosing by augmenting the program to include one-on-one lifestyle coaching with CHWs. We will also focus the program primarily on dietary behaviors by eliminating Alive!’s physical activity intervention component. Several RCTs have shown that the Alive! platform is effective at improving dietary habits, physical activity, body weight, and glycemic profile [16, 32, 33] in a dose-dependent manner, and CHWs have demonstrated their ability to support digital interventions targeting CVD [38].

Cultural adaptation of Alive! will occur during the first 12 months of this proposal and will involve formative development and refinement of our intervention. In partnership with NutritionQuest developers, we will integrate CBPR to culturally adapt Alive! using processes that include focus groups with church members and community partners, which will inform the design and content on the web-based platform including the incorporation of motivational testimonials and spiritual messaging. For messaging, we will use an intersectional approach that accounts for heterogeneity within AA populations (e.g., age—45-years vs 75-years—and gender differences). This will be an iterative process of changes and revisions using a feedback loop and a narrative performance scale (NPS) [39]. The NPS is a validated tool used to measure the effect narrative health messages on the behavioral intent of a targeted individual. We will utilize the Ecological Validity Model [40], which includes 8 elements for adaptation (language, persons, metaphors, content, context, concepts, goals, methods), allowing researchers to (1) linguistically tailor the intervention by focusing on colloquial language and limiting medical jargon; (2) peripherally tailor the intervention by focusing on presenting materials in a manner that is culturally appealing; and (3) socio-culturally tailor the intervention by contextualizing health issues in the broader social context such as structural racism and discrimination while imparting the importance of a health behavior [41]. Specific Alive! modules for cultural targeting included (1) the small-step recommended goals tailored to the individual’s dietary assessment results, which are recommended to participants each week; (2) “Health Notes” health education articles that participants are expected to review weekly; and (3) infographics and media displayed to the participant on their Alive! homepage and in emails.

Participants within churches randomized to the intervention arm (SBIRT) will be enrolled into the CAP. Participants who cannot afford to modify their diets are referred to our CBO network specializing in providing healthy food to food insecure residents and computer tablets are provided by the study to participants without uninterruptible access to a web-based platform from a computer or smartphone (determined from baseline assessments). The project coordinator (PC) will be available to provide additional technical assistance even though studies of an unmodified version of Alive! have shown good functional feasibility among AA populations [37]. Following SBIRT sessions, CHWs will introduce the CAP program. We will assess participants for food insecurity using the six-item short form food security survey [42]. Enrollment in Alive! is supported by CHWs. This involves creating an online account using the participant’s name and email and completing embedded assessments. CAP will maintain the behavior change approaches in the unmodified version of the Alive! intervention, which uses information derived from assessments to personalize goals. Every week, participants will be expected to choose at least one eating goal, read two short Health Notes, complete quizzes associated with the health education material, and track parameters related to their dietary behaviors. They will earn virtual points for meeting goals redeemable for small non-monetary study incentives. CHWs will supplement each participant’s lifestyle programming with 1:1 coaching delivered in 15- and 30-min sessions by telephone/zoom once-a-week for 4 weeks and every 2 weeks for 8 weeks. Sessions will focus on providing encouragement around small step weekly goals. Fidelity procedures similar to Aim 1 will be implemented. Eating habit assessments within Alive! will be captured by a brief FFQ modified to include foods that can increase or reduce inflammation. Alive! software is programmed to generate an inflammatory diet report in real time for each participant from the brief FFQ. Alive! will also generate the Dietary Inflammatory Score (DIS) [43] which is based on food group intake (e.g., fruits), unlike the older Dietary Inflammatory Index (DII) which is based on nutrient intake (e.g., carbohydrates). This makes the DIS more useful for intervention studies compared to the DII [43, 44]. Finally, we will develop a participant level “dashboard” for CHWs with summarized data on lifestyle and dietary factors which will be used by CHWs to tailor their one-on-one coaching. This dashboard will be secure and HIPAA compliant.

#### Referral as usual

Distributing cancer prevention pamphlets during organized church health fairs is a common health education approach. We will adopt a similar approach and distribute CRC brochures promoting the new CRC screening guidelines to participants randomized to usual care. Brochures will be distributed by CHWs to participants after baseline screening. For consistency, we will utilize one brochure from the National Cancer Institute and one from the Center for Disease Control and Prevention (CDC). CHWs will also distribute a list of local CRC screening referral sites (GI clinics) and a community resource guide to study participants. The Directory will function as a primary referral source to CRC screening. No CRC screening referrals will be made in this arm. At 6-month follow-up, participants will be asked by RAs whether they had a colonoscopy or any other screening test for CRC.

### Criteria for discontinuing or modifying allocated interventions {11b}

There are no preplanned reasons for modifying or discontinuing the intervention.

### Strategies to improve adherence to interventions {11c}

The strategies we will utilize in this study to increase adherence are innovative in the following ways:

1. *Strategy of the intervention:* MI has been shown to increase colorectal cancer screening [45]. We employ a trauma-informed evidence-based approach utilizing MI called Screening (with a validated instrument), Brief Intervention (using Motivational Interviewing), and Referral to Treatment (SBIRT) originally designed for mental health behaviors [23] for CRC screening and referral. CHWs in the intervention arm will utilize MI to decrease ambivalence and increase motivation for treatment.
2. *Church-placed setting*: We adopt a “meet people where they are” approach by integrating our intervention into the church setting from where referrals will occur.
3. *Use of CHWs:* CHWs can reduce health system mistrust and allow us to build health promotion capacity in the church and broader community.
4. *Adaptation of Alive*!: We are culturally adapting Alive! [33], an existing NCI EBCCP listed web-based program to target CVD and CRC risk.

### Relevant concomitant care permitted or prohibited during the trial {11d}

There are no concomitant care permitted or prohibited during the study.

### Provisions for post-trial care {30}

This study is classified as minimal risk, and therefore, there are no anticipated harm to the participants as a result of participating in this study. There are no plans for direct provision of post-trial care.

### Outcomes {12}

This cluster RCT will evaluate the impact of a CHW enhanced CRC screening intervention on screening uptake (clinic-based colonoscopy or home-based stool test) (primary outcome). The definition of screening uptake is the subject’s self-report of completing a CRC screening test plus the research team’s verification of this completion from medical records. The secondary outcomes include the effect of the CAP program on DIS and on changes to CVD risk captured by LS7 scores. Secondary outcomes will be measured using a modified Block Food Frequency Questionnaire (FFQ) [46] and Life’s Simple 7 measure, respectively. The FFQ contains a food list of 83 food items that asks about eating habits in the past month, provide estimates of dietary intake, and rank individuals along the distribution of intake. Pictures are provided to enhance accuracy of quantification of portion sizes. The FFQ is intended for either self- or interviewer-administration and has been validated in studies [35]. RAs will collect FFQ data at baseline, 3, and 6 months. The LS7 measure is based on 7 domains: 3 health physiological metrics (glucose, cholesterol, and blood pressure levels) and 4 health behavior-related metrics (body mass index, physical activity levels, diet quality, and cigarette smoking). Each factor is comprised of items that are rated at “ideal,” “intermediate,” or “poor,” corresponding to a score of 2, 1, or 0 respectively. LS7 scores range from 0 to 14 and are calculated from the composite of the factor scores. CVD health is then classified as inadequate (0–4), average (5–9), or optimum (10–14). Achieving a greater number of ideal LS7 metrics is associated with lower risk of dying after stroke and all cause cardiovascular mortality in a dose-dependent manner [47, 48]. RAs will collect LS7 measures at baseline, 6 months, and 1 year in the church with standard approaches and FDA approved validated point of care testing (POCT) devices for glucose and cholesterol.

### Participant timeline {13}


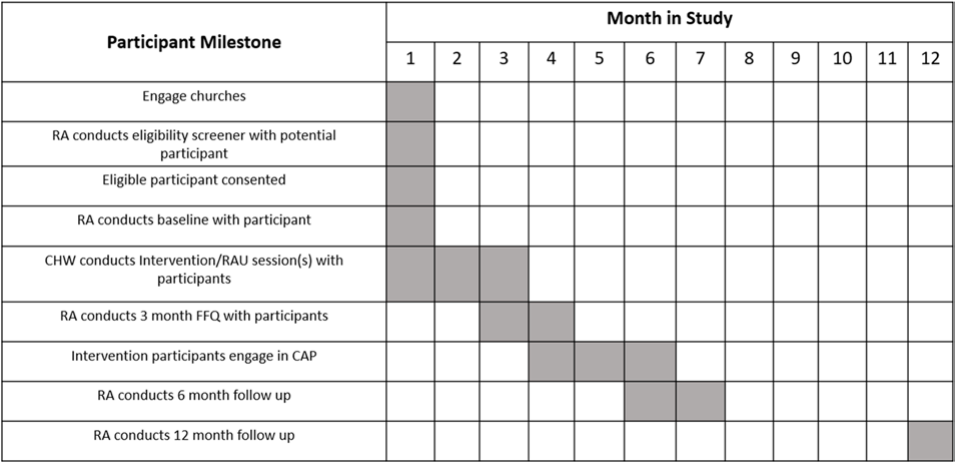


### Sample size {14}

We will conduct a two-arm, cluster RCT in 22 Black churches. From each of these 22 churches, we will recruit 20 subjects for a total sample size of 440 subjects. Based on our prior study in which approximately 50% of AAs above age 50 years were not up-to-date with screening [49], we will screen at least 40 adult churchgoers aged 45 years and older per church for study eligibility (total screening = 880 subjects).

### Recruitment {15}

#### Recruitment of community health workers

Certified CHWs (two from each church for a total of 44) trained through the Columbia InTOuCH program will be recruited from our existing cohort of 212 CHWs. The Columbia InTOuCH program trains 45 CHWs per year on an ongoing basis, which will further expand our recruitment pool. Interested CHWs will be interviewed to assess their experience working with people to get screened for CRC and reasons for participating, as well as to clarify the roles and expectations of CHWs. All study CHWs (*n* = 44) will receive an additional 18 h of booster training sessions guided by the information-motivation-behavioral skills (IMB) model [50]. CHWs will complete measures of knowledge (CRC and CVD), Motivation, and Behavioral Skills, including Self-Efficacy for screening and coaching activities. Experts will deliver training modules covering (1) CRC treatment, prevention, disparities, and screening guidelines; (2) Inflammatory Diet and related CVD risk factors; (3) SBIRT procedures; (2) Alive! procedures; and (3) Human Subjects protections (CITI and HIPAA).

#### Recruitment of churches and participants

Two CHWs and at least one Project Coordinator (PC) will introduce the CRC study to potential churches through informational sessions, and CRC awareness forums. CHWs will engage community members at screening events and gauge their interest in participating in the study. A QR code will be found on the study flyer that links to a Qualtrics sign-up sheet which the CHWs will administer. The form will be used to obtain participant demographic information. If the participant indicates they are interested in getting screened, the form will prompt the participant to provide their contact information, allowing a research assistant to contact them at a later time to be screened. Responses to this survey will be kept confidential. Only the principal investigators and the study staff will be able to see the results of the sign-up sheet. All data obtained from response to this form will be stored in the secure CUMC Qualtrics platform.

#### Recruitment of clergy

The CHWs and PC will present the study process and information on the semi-structured interview or focus groups to the lead pastor, and clergy members.

#### Recruitment of clinical providers

Providers will be identified from the study’s network of local GI clinics based on their capacity to provide CRC screening opportunities for study participants. The study PI and project director (PD) will connect with potential providers over email and zoom to discuss options for a partnership. Once a partnership is established, the study staff will work closely with clinical providers to develop a process for referring study patients to the hospital for CRC screenings.

## Assignment of interventions: allocation

### Sequence generation {16a}

In this study, randomization will occur on the church level to reduce chances of contamination through interactions between congregants in the same church. Four churches will be randomized in year 1; six churches in year 2; six churches in year 3; and six churches in year 4 to reach our target sample size of 22 churches. Churches will be matched based on size (number of members) and randomized in a 1:1 ratio to either SBIRT and ALIVE! or RAU. A randomization sequence will be generated by the study statistician according to CONSORT guidelines. Randomization will occur on the church level prior to the start of screening events to allow for assignment to specific booster trainings with the CHWs. At the end of data collection, churches assigned to the RAU arm will be offered SBIRT.

### Concealment mechanism {16b}

The randomization procedure will be conducted by a biostatistician who then passes the assignment directly to the Study Director. The Study Director will notify community health workers of their church assignments. The data coordinating center (DCC) study liaison will be the only additional person notified of the randomization assignments.

### Implementation {16c}

At each screening event at the churches, two CHWs and at least one RA will be present, in addition to either the project coordinator or study director. The team will assist with conducting eligibility screenings, scoring screeners, and enrolling eligible participants into the study. Following the screenings, RAs will schedule a time to meet with the participant for the baseline within the next week after screening and consent.

## Assignment of interventions: blinding

### Procedure for blinding {17a}

All research assistants collecting data at all waves will be blinded.

### Procedure for unblinding if needed {17b}

There will be no circumstances in which unblinding will be necessary.

## Data collection and management

### Plans for assessment and collection of outcomes {18a}

The primary outcome measure, an adapted version of the Behavioral Risk Factor Surveillance System (BRFSS) CRC screening tool, and the DIS will be collected at 6 months. The secondary outcome LS7 will be collected at 6 and 12 months. Table 1 outlines the measures used to construct the baseline, 3-month, 6-month, and 12-month assessments. Major timepoints in the study protocol are outlined in Table 2.

**Table 1:** Clinical measures

Main measures	Baseline	3 months	6 months	12 months
	**Physiological measures**			
Biometric Life Simple-7 (blood pressure, A1C, BMI, and cholesterol)	X		X	X
	**Self-report measures**			
Sociodemographics	X		X	
NIMHD PhenX Social Determinants of Health	X		X	
Health Literacy	X		X	
Everyday Discrimination Scale	X		X	
Thompson BQ Healthcare Trust Scale	X		X	
Colorectal Cancer (CRC) Screening Measure	X		X	
Verification CRC Screening Completion			X	
Perceived benefits and barriers of CRC screening	X		X	
Food Frequency Questionnaire	X	X	X	X
Six-Item Food Insecurity Survey	X		X	
CDE Health conditions	X		X	
Health Literacy Newest Vital Signs	X		X	
Perceived Barriers and Benefits	X		X	
Perceived Stress Scale	X		X	
Physical Activity	X		X	X

**Table 2:**
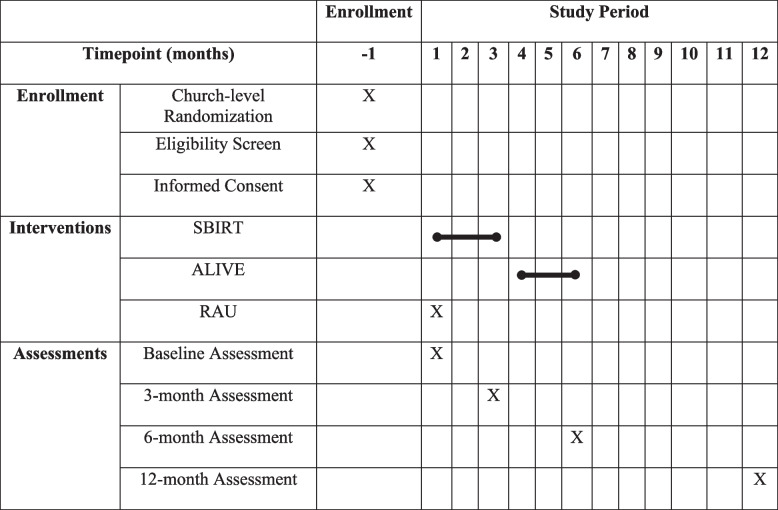
SPIRIT Schedule of Enrollment, Interventions, and Assessment

#### Identify contextual factors that act as facilitators or barriers of CRC screening update (secondary outcome)

We will employ a concurrent, convergent mixed-methods research design [51] to gather multi-level factors that influence both delivery of the intervention and its impact on key health behaviors. We will build off of the CFIR [18]. Data collection and analysis will be conducted concurrently (QUAN + QUAL) [51] to understand effectiveness and implementation outcomes using a more nuanced, in-depth approach. First, after reaching the 6-month follow up point, we will use the CFIR as a guide to conduct semi-structured interviews or focus groups across each level of the Socio-Ecological Model: individual [randomly selected CRC screening guideline discordant subjects across the usual care and intervention groups (*n* = 22 of each for total of 44)], interpersonal [CHWs (*n* = 44)], institutional [clergy from each site (*n* = 22)], and community [clinic providers (*n* = 11)]. These interviews will enable a deeper understanding of the multi-level barriers and facilitators to behavior change and screening uptake across CFIR domains (policy, organizational/church, implementer, intervention, implementation processes) that impact delivery of the intervention; we will apply a recently adapted version of this framework that employs a race-conscious frame to CFIR constructs and domains to understand ways that structural racism interacts with intervention implementation and uptake [17]. Additionally, to enhance understanding of the mechanisms through which the intervention works (e.g., how and why?), CHWs will complete brief surveys about their role commitment, self-efficacy, organizational, and role-related benefits/challenges, informed by our extensive former work on successful implementation of CHW programs in AA community settings and churches [52, 53]. Each pastor will also complete the Faith-Based Organization Capacity Inventory (FBO-CI) [54] interview to assess their church’s health promotion experience and research capacity, which allows us to explore whether there are differences in impact and implementation of the intervention based on these characteristics. Implementation data will be collected on key indicators from the RE-AIM evaluation framework from implementation science, including Reach—the absolute number of congregants willing to participate; Adoption—the percentage of churches that employ 12 CRC Screening identification events in a year; and Maintenance—the extent to which CRC screening referrals continue to be delivered 1-year following the end of the data collection period in the absence of research support [55, 56]. This information will help identify significant challenges or inequities along the implementation continuum. All interviews are conducted by trained RAs either in person or over the telephone.

### Plans to promote participant retention and complete follow-up {11c}

#### Plan for church and participant retention

Each church will receive a $1000 donation for their time and space. Pastors will sign an agreement that commits their church to (1) identify a church champion to assist CHWs implement screening days and (2) host at least 12 CRC screening drives per year. We will also implement proven retention strategies for AA participants such as regular telephone contact to remind participants of upcoming study appointments; toll-free study telephone number for contacting study staff; diverse study staff; and direct assistance with transportation to study visits. Participants will receive an incentive of $50 for completion of baseline, 6-month, and 1-year measures and $25 for completing the 3-month FFQ.

#### Plan for CHW retention

We have a successful retention program for our CHWs that has led to an 85% retention of our CHW cohort. The program includes recreational engagement and relationship building; continuous health education; and mindfulness training. CHWs will meet monthly to discuss challenges and will have 24-h access to PIs via cellphone.

### Data management {19}

This trial will partner with the DCC which will be responsible for the following: (1) development of a computer-based data collection system, (2) training staff and data collectors, (3) randomization, (4) monitoring data and quality control, (5) data processing, and (6) data analysis. Study milestones and progress will be monitored by the DCC. Study progression will be reported through regular reports.

All screening and assessment data will be collected using a REDCap form which will increase the accuracy of the data collected as responses will be limited to pre-provided values. Scoring and cleaning processes will be developed by the DCC for scales within instruments. This cleaning process will provide an additional layer of protection to double check the accuracy of the data. The DCC project manager will be responsible for periodically reviewing the data collected to ensure there are no duplicate records, incorrect collection dates or times, or out of range values. Following any corrections made, the project manager will review the items to ensure no anomalies remain. Quality assurance measures will include periodically reviewing entire files.

Data collection will be completed on secure laptops and office desktop computers that are password protected and encrypted. Laptops and hardcopies of data will be stored in a locked storage area on site.

Electronic data will be backed up daily or weekly to a backup server at the DCC. Additional backup hard drives will be securely stored in a fireproof safe. Protected Health Information (PHI) will be stored in a secure device that is not connected to the internet. All devices will be password protected and their drives will be encrypted. The network will be protected against hackers and unauthorized internet access though a hardware-based firewall device, which will also serve to filter out spam. Devices will be protected against malware using anti-virus software that is automatically updated through “push-technology.”

### Confidentiality {27}

#### Patient information

Participant confidentiality will be protected in multiple ways. Specifically, participant data collected following consent by participants will be protected. RAs will collect, gather, and enter required data onto study data forms. Screened participants who do not meet study eligibility will only have specific screening data entered into the study database. The collected data will include gender, age, race, and reason for exclusion. When personal data, e.g., telephone numbers, email address, and home addresses—are collected, they will be stored on password-protected computers with secure servers and firewalls. The information will only be available to IRB approved study personnel who are required to take HIPAA and CITI training. Of note, all CHWs in this study will be required to take HIPAA and CITI training as part of their on-boarding process to participate in this study. PHI will be removed from the study database upon study completion. All data obtained from this study will be used for research purposes only and will comply with Federal HIPAA regulations. These data will be de-identified using a unique identifier whose code is only known to research staff and stored in a secure encrypted electronic database such as REDCAP.

#### Paper assessments and files

The Project Coordinators will prepare and maintain a participant-specific binder for each participant containing all case report form (CRF) records. A regulatory file will also be maintained to include the IRB-approved Protocol, original Informed Consent documents, HIPAA forms and other study-related regulatory documents. All paper research records and CRFs will be maintained in a locked file cabinet in a secure facility within the Department of Neurology. Access to the research records and study database will be restricted to study personnel as approved by the MPIs and IRB. As with all studies conducted at Columbia University, this study is also eligible for a random audit by Columbia University’s Office of Compliance.

#### Electronic assessments

This study will use Research Electronic Data Capture (REDCap) for data capture and management.

#### Secure data transfer procedures

Statisticians at the DCC will download data from assessments from the Columbia secure server through VPN connections.

#### Data access

Access to data will require two steps. First, the user must log into the University VPN after which they will be directed to log into the Columbia file server. Study personnel will reset their passwords every 6 months and be required to use password-protected laptops/computers. The data will be kept on HIPAA compliant and encrypted servers that will only be accessible to IRB-approved personnel.

Further efforts to ensure participant confidentiality will include the following:

- Study data will be linked with a unique code number assigned to each study participant
- Participant names, code numbers, and study data will be stored in a locked folder that can be accessed only by study staff
- Information kept on the computer will be coded numerically
- All study data will be reported in a group format with no individual data presented
- Records will be made available only to research personnel, and Federal, State, and Institutional staff, who may review records as part of routine audits
- Confidential subject records may be accessed by legal advocacy organizations with authority under State law, but this information cannot be re-disclosed without participant consent.

### Plans for collection, laboratory evaluation, and storage of biological specimens for genetic or molecular analysis in this trial/future use {33}

To assess Aim 3, blood will be obtained from participants through a finger stick test using a point of care device. No biological specimens will be stored for genetic or molecular analysis in this trial/future use. RAs will collect all finger stick measures in a private space within the church setting following all safety precautions.

## Statistical methods

### Statistical methods for primary and secondary outcomes {20a}

#### Power analysis for Aim 1

This aim compares the effect of the SBIRT to RAU on CRC screening uptake. It is hypothesized that the intervention will lead to increased CRC screening (colonoscopy or stool-based test—the primary outcome) compared to RAU at 6 months. An intent-to-treat (ITT) set of analyses is proposed, with the alpha level set at 0.05. The sample size is based on the number of subjects needed to provide adequate power to detect the hypothesized group differences in the primary outcome, adjusted for clustering within churches. The comparisons of SBIRT to RAU on the guideline-concordant CRC screening rate will be conducted using a logistic regression model:

$logit\left(p\right)={\text{log}}\left(\frac{p}{1-p}\right)= {\beta }_{0}+{\beta }_{1}X+{\tau }_{c}$, where *X* is a binary variable (0 for usual care and 1 for SBIRT intervention), ${\tau }_{c}$ is a random effect for clustering within churches, and *p* = prob(Y = 1, CRC screening rate). The hypothesis H_0_: ${\beta }_{1}=0$ vs H_1_: ${\beta }_{1}={\beta }^{*}$ will be tested with the formula for determining the total sample size. [57–60]: ${n}^{*}=\frac{{(V{(0)}^{1/2}{Z}_{1-\alpha /2}+V{{(\beta }^{*})}^{1/2}{Z}_{1-\beta })}^{2}}{{P}_{1}{\beta }^{*2}}(1+2{P}_{1}\delta )$ with ${\beta }^{*}=log\frac{{P}_{2}(1-{P}_{1})}{{P}_{1}(1-{P}_{2})}$, with $V\left(0\right)=\frac{1}{1-B}+\frac{1}{B}$, $V\left({\beta }^{*}\right)=\frac{1}{1+B}+\frac{1}{Bexp\left({B}^{*}\right)}$, $\delta =(V{\left(0\right)}^{1/2}+V{\left({\beta }^{*}\right)}^{1/2}R)/(V{\left(0\right)}^{1/2}+V{({\beta }^{*})}^{1/2})$, $R=v\left({\beta }^{*}\right)B\left(1-B\right){\text{exp}}\left(2{\beta }^{*}\right)/{\left(Bexp\left({\beta }^{*}\right)+\left(1-B\right)\right)}^{2}$, where ${P}_{1}$, ${P}_{2}$ are the CRC screening rates of usual care and SBIRT (intervention), respectively, and B is the proportion of the sample assigned to each of two arms (*B* = 0.5), δ is a small rate adjustment, when ${P}_{1}<0.2$ (δ = 0 when ${P}_{1}\ge 0.20$), adjusting for unreliability and clustering (*g* = 1 + (*s* − 1)*icc):$n={n}^{*}g/{R}_{el}$. The assumptions are as follows: for *α* = 0.05 (2 tailed test), 1 − *β* = 0.80 (power), ${R}_{el}=0.95$ (adjusted for unreliability), *g* = 1.57 (variance inflation factor adjusting for clustering), with cluster size *s* = 20 and icc (intracluster correlation coefficient = 0.03). ${P}_{1}$ (*X* = 0, usual care group), ${P}_{2}$ (*X* = 1, intervention group), *B* = 0.5, *n* = total sample size, and power for *n* = 396 (*m* = 198 per group, about 11 churches per group with attrition rate = 10% at 6 months with an average of 20 participants per church)/

Regarding the CRC screening rate, a sample size of 198 subjects per group (10 to 12 churches per group, average of 20 subjects per church, attrition rate of 10% at 6 months) is required to provide 80% or greater power to detect a group difference of 15% (a clinically meaningful increase) in the rates of completed colorectal cancer screening at 6 months. Thus, power will be sufficient to detect the hypothesized difference from baseline in both arms. As shown above, these calculations assume a baseline rate of 0 and scenarios with increase in screening rates in the usual care group from 0.20 to 0.50. Thus, with an overall sample size of 440 subjects or 220 per arm (11 churches per arm with 20 subjects in each church), the study is powered adequately, even with attrition. Based on our prior study in which approximately 50% of AAs above age 50 years were not up to date with screening [49], we will screen at least 40 adult churchgoers aged 45 years and older per church for study eligibility (total screening = 880 subjects).

#### Power analysis for Aims 2 and 3

Aim 2 is to evaluate the effect of a CAP incorporated into the intervention arm on DIS at baseline and 6-month follow-up, and Aim 3 is to evaluate the effect of CAP on changes in LS7 at baseline, 6 months, and 12 months. Sensitivity analyses will be performed including data from the 3-month wave. Power calculations were performed for an analysis of covariance (ANCOVA)-type model: ${y}_{ijk}={\mu }_{jk}+{y}_{i0}+{\tau }_{c}+{e}_{ijk}$, where ${\mu }_{jk}$ is the group mean for follow-up DIS and LS7, ${y}_{i0}$ is the baseline measure, *j* = 1,2 follow-ups and *k* = 0, 1 for group, and ${\tau }_{c}$ is a random effect for clustering within church. The power analysis equation for one follow-up is: $n=\frac{2{\left({z}_{\alpha }+{z}_{\beta }\right)}^{2}{\sigma }^{2}\left(1-{\rho }^{2}\right)g}{{d}^{2}{R}_{el}}$: where $\rho$ is the correlation between the outcome and baseline values, $\sigma$ is the standard deviation, ${d}_{I}$ is the follow-up difference between 2 groups, ${R}_{el}$ is the reliability of the outcome, adjusting for clustering within churches, g = 1 + (*s* − 1)*icc, and *n* is number of subjects per group.

The power analysis equation for 2 follow-ups examined in Aim 3 is $m=2g{\left({z}_{\alpha }+{z}_{\beta }\right)}^{2}\sigma (1-{r}_{c}^{2})\sum \sum {r}_{ij}/{(R}_{el}\sum {d}_{i}{)}^{2}$ and ${r}_{c}=\sum {r}_{i1}/\sqrt{\sum \sum {r}_{ij}}$, where *g* is the clustering adjustment for church, ${r}_{ij}$ is the correlation matrix for 2 follow-ups, ${r}_{i1}$ is the correlation between baseline and 2 follow-ups, and *d*_*i*_ is the follow-up difference between 2 groups for two follow-ups, with *i* = 1,2 and *j* = 1,2. The correlation between the baseline and 2 follow-ups (half year, 1 year) will be modeled as CS (compound symmetry (constant: ${r}_{i1}=\rho , \rho$)) and AR_1_ (Autoregressive (Exponential decay, ${r}_{i1}=\sqrt{\rho }, \rho$)). The difference between the 2 follow-ups will be modeled as unequal: ${d}_{i}$=(¾*d*, *d*)—the half year follow-up difference between 2 groups is ¾ of the 1 year study-end difference; and equal: ${d}_{i}$=(*d*, *d*)—the two follow-up group differences are the same. The minimal detectible effect size (expressed as Cohen’s *d*) with *m* subjects per group was calculated assuming: *α* = 0.05 (two-tailed), ${R}_{el}$(reliability), *g* = 1.57 (clustering adjustment for church as descripted above), *m* = 198 per group at 6 month follow-up assuming at least 2 waves of data and an intent-to-treat (ITT) model for the third wave (220 subjects at baseline, 10% attrition at 6-month follow-up). The correlation between baseline and 1 year follow up is assumed to be 0.5, 0.6 and power = 80%. Two scenarios regarding the covariance structure and the difference between groups were also examined.

As regards the dietary inflammation score (DIS), for the DIS outcome, with baseline and one follow up, it is assumed that *σ* = 1 for the standardized score. Assuming *α* = 0.05 for a two-tailed test, 10% attrition, with *m* = 198 per group at 6 months (220 at baseline, 11 churches per group), adjusting for reliability = 0.8, and the clustering effect of church, it will be possible to detect a small effect size of *δ* = 0.303 (Cohen’s *d* = 0.303), with power of 0.80. With the correlation between baseline and follow-up equal to 0.6, Cohen’s *d* = 0.280 (an effect size of *δ* = 0.280).

For the LS7 outcome, with baseline and two follow-ups, it is assumed that *σ* = 2.7 (standard deviation). Assuming the difference between the 2 groups is unequal will result in lower power than if equal. A correlation structure of compound symmetry will result in lower power than autoregressive (AR). The primary analyses assume ITT for the 3rd wave, *α* = 0.05 for a two-tailed test. However, effects assuming 20% attrition, with *m* = 176 per group at 1 year study-end were also calculated (baseline *m* = 220, 6 months m = 198, and 1 year m = 176). With an *m* = 198 per group at 6 months (ITT model for 3rd wave), it will be possible to detect an effect size of *δ* = 0.764 (Cohen’s *d* = 0.283), with power of 0.80. With the correlation between baseline and follow-up equal to 0.6, Cohen’s *d* = 0.265 (which translates to an effect size of *δ* = 0.716). Examining different scenarios regarding the difference between the groups and the correlation structure of compound symmetry, or AR, the detectable effect sizes range from *δ* = 0.645 (Cohen’s *d* = 0.239) to *δ* = 0.810 (Cohen’s *d* = 0.300) with power of 0.80.

#### Analysis for Aim 1

Logistic-type regression analyses will be used to test the hypothesis that those assigned to SBIRT will have significantly higher CRC screening rates compared to those randomized to RAU at 6 months. Clustered sampling effects due to churches will be incorporated using a multilevel modeling approach. Ideally, the randomization of participants to treatment arm and the absence of significant selection and/or attrition biases will obviate the need for inclusion of covariates. In the event that the logit analyses indicate one or more sources of potential bias, the predicted values of those analyses will be included as covariates in a multilevel logistic regression model (SAS, PROC GLIMMIX).

#### Analysis for Aims 2 and 3

An ANCOVA-type model, using SAS Proc Mixed will be used to allow for flexible modeling of assumptions, treatment of missing data and inclusion of all subjects with at least one wave of data. The basic model is ${Y}_{i}={\beta }_{0}+{\beta }_{1}{Y}_{0i}+\beta {X}_{i}+{e}_{i}$. Based on prior analytic experience with the outcome variables, it is not expected that transformations will be necessary; however, distributions will be examined for confirmation. The continuous longitudinal outcomes will be modeled as functions of baseline values, randomization groups, covariates, and interactions as necessary. Prior to analyses, baseline values of all variables will be examined. Variables differing between the groups will be examined in secondary analyses.

### Interim analyses {21b}

This study does not have pre-planned interim analyses or stopping guidelines.

### Methods for additional analyses (e.g. subgroup analyses) {20b}

No additional subgroup analyses are preplanned.

### Methods in analysis to handle protocol non-adherence and any statistical methods to handle missing data {20c}

Examination of baseline differences on key variables between completers and lost-to-follow-up will be conducted to inform the nature of missing longitudinal data. Methods of examining missing data, e.g., propensity scores, EM algorithm, and multiple imputation sensitivity analyses will be considered if necessary. A specific imputation approach, e.g., Markov Chain Monte Carlo will be used, depending on the amount and pattern of missing data. SAS Proc Multiple Imputation and MIAnalyze will be used for these analyses.

### Plans to give access to the full protocol, participan-level data, and statistical code {31c}

The full study protocol is available on clinicaltrials.gov—NCT05174286. The data analyzed will be made available on reasonable request to the corresponding author.

## Oversight and monitoring

### Composition of the coordinating center and trial steering committee {5d}

The coordinating center and trial steering committee include the following members: the Contact-Principal Investigator (Columbia University, Dept. of Neurology) will be responsible for corresponding with NIH. In addition to providing timely reports to NIH, regarding (i) Unanticipated problems or unexpected serious adverse events that may be related to the study protocol, (ii) IRB-approved revisions to the study protocol that indicate a change in risk for participants, and (iii) notice of any actions taken by the IRB or regulatory bodies regarding the research and any responses to those actions, the contact PI will be responsible for reviewing all CRC screening questionnaires and assessments by participants at baseline, 3, 6, and 12 months; and reviewing rates of participant referral to treatment.

The Multiple-Principal Investigator (Ichan School of Medicine at Mount Sinai) will provide site oversight by auditing CHW protocols and church sites where the intervention and control programs will take place. In addition to intervention fidelity monitoring, the co-PI will perform random interviews with CHWs (at least once at each church site) to evaluate the presence of any adverse reactions to the curriculum that may not be captured by questionnaire data.

The DCC will regularly review program data, which will be discussed with the MPIs monthly. The Project Director (PD) will be present on site at every intervention and control program. In addition to project management activities such as recruitment, consenting, training, and data collection, the PD will be responsible for identifying and reporting any adverse encounters—related or unrelated to the intervention—to the Multi-PIs. These include adverse emotional responses, interpersonal conflicts, physical accidents, or any other participant safety concerns that may occur during the intervention.

### Composition of the data monitoring committee, its role and reporting structure {21a}

#### Data safety monitoring plan

In compliance with NIH requirements, we will establish a data and safety monitoring plan (DSMP). The purpose of these plans is to ensure the safety of participants and the validity and integrity of the data. Considering the study rationale, population, procedures, and the risk: benefit profile as outlined; the overall risk level for participation in this screening intervention is classified as minimal. Due to the classification of this study as minimal risk, the members of the investigative team described below will serve as the Data Safety Monitoring Committee and will perform the monitoring.

### Adverse event reporting and harms {22}

Diligent study safety monitoring will be conducted by the co-Investigators outlined in the monitoring entity section. Although the risks associated with this research is minimal, we will monitor the effects of the study on adverse participant emotions related to distress or stigma from being questioned about personal mental health concerns. The Project Coordinator / Director will identify adverse events through direct observation and survey response data.

The Project Coordinator will review the circumstances to determine whether institutional notification is necessary. The study Contact-PI will assume reporting responsibilities for all adverse events to the Columbia University IRB and NIH, which have the authority to halt the trial if it perceives that harm is occurring due to the intervention. Additionally, per Columbia’s IRB policy, all participant deaths, protocol deviations, complaints about the research, and breaches of confidentiality are reportable events.

### Frequency and plans for auditing trial conduct {23}

#### Monitoring study safety

The data and safety monitoring plan for this study will include reporting adverse events to the IRB and NIH trials. The Columbia IRB will have the authority to halt the trial if it believes that harm is occurring as a result of the intervention. All non-time sensitive adverse events will be reported to the NIH through yearly progress reports and the final report submitted at the end of year 5 of the study. All study staff will complete the Good Clinical Practice and Human Subject Protection trainings prior to working with participants. Internal monthly quality control audits, periodic assessments of data quality and timeliness, participant recruitment, accrual and retention, and protocol fidelity monitoring will also be executed. During the early stages of enrollment, one or more “Early Safety/Trial Integrity Reviews” will be held to review safety considerations and ensure proper implementation.

### Plans for communicating important protocol amendments to relevant parties (e.g., trial participants, ethical committees) {25}

All protocol amendments will be submitted to the Columbia IRB and if applicable, participants will be notified. Study personnel will submit annual progress reports to the NIH (funder). Breach report forms will be completed to document any deviations from the original study protocol.

### Dissemination plans {31a}

#### Community dissemination

A dissemination symposium will be organized by the Community Coalition at a community-based venue in Harlem. The investigators and selected members of the Coalition will co-present. The symposium will be specifically organized to gather community feedback. The participating churches and stakeholder organizations will have access to the content of the symposium.

#### Study dissemination and implementation

If the SBIRT and ALIVE! interventions are shown to be effective through this trial, our next step will be to design a Hybrid Type 3 Implementation-Effectiveness Study to disseminate and implement the intervention across the community of Black Churches throughout New York State.

## Discussion

Deaths from CRC have steadily declined, although a shift in the patient population towards younger ages has been observed [61]. Disturbingly, recent trends reveal a 2% increase in incidence and 1% increase in death rates among people younger than 50 years [4]. Today, 94% of new cases occur in adults 45 years or older, [62] and by the year 2030, CRC will become the leading cause of cancer-related deaths among those aged 20–49 [63]. Data such as these led the American Cancer Society to lower the recommended age for CRC screening tests from age 50 to age 45 years, while people at high risk (e.g., significant family history) for CRC should consider screening tests before age 45 years. In May 2021, the US Preventative Services Task Force (USPSTF) adopted the lower age recommendations [3].

Black churches can increase the uptake of evidence-based screening for cancer [64] and cardiovascular disease [38]. In urban AA communities, 65–80% attend church regularly [65], many of whom are exposed to a church “Health Ministry” of volunteers who provide health education, screening, and resources to community members [66]. Church-based interventions include the use of digital health applications, which have been conducted successfully and with low attrition rates [38].

CHWs are trusted community members [67] trained to function as health educators, health coaches, navigators, care coordinators, case managers, and patient advocates [68]. CHWs can serve as linkages to clinical care and reduce barriers related to SDoH including transportation, childcare, and health system navigation. They can support chronic disease detection and management [69, 70] and increase cancer screening rates [71, 72] in a cost-effective manner. We hypothesize that utilizing CHWs for church-based colorectal cancer screening can support chronic disease detection and management and increase cancer screening rates in a cost-effective manner.

## Trial status

Protocol version IRB-AAAT9307, May 2, 2023. Recruitment for this study began in March 2023 and is expected to be completed in 2027.

## Data Availability

Study-related data analyses will be completed by the Data Coordinating Center. All guidelines for sharing data will be followed. Plans for archiving and sharing data will be finalized within 2 years of the end of the study.

## Abbreviations

CRC: Colorectal cancer
U.S.: United States
AAs: African Americans
CHWs: Community health workers
SBIRT: Screening, Brief Intervention using Motivational Interviewing, and Referral to Treatment
LS7: Life Simple 7
CVD: Cardiovascular disease
CFIR: Consolidated Framework for Implementation Research
FBO-CI: Faith-Based Organization Capacity Inventory
FIT: Fecal immunochemical test
gFOBT: Guaiac fecal occult blood test
sDNA: Stool DNA test
CT: Computed tomography
RAU: Referral as usual
CAP: Culturally adapted ALIVE! Program
CBPR: Community-based participatory research
RAs: Research assistants
TIC: Trauma-informed care
MI: Motivational interviewing
OARS: Open-ended questions, affirmations, reflections, and summary statements
GI: Gastrointestinal
RE-AIM: Reach, effectiveness, adoption, implementation, maintenance
FFQ: Food Frequency Questionnaire
DIS: Dietary inflammatory score
POCT: Point of care testing
PC: Project coordinator
DCC: Data coordinating center
CRF: Case-report form
ITT: Intent-to-treat
PD: Project director
DSMP: Data and safety monitoring plan
USPSTF: US Preventative Services Task Force
NCI: National Cancer Institute
EBCCP: Evidence-Based Cancer Control Programs
DIS: Dietary Inflammatory Score
DII: Dietary Inflammatory Index
IMB: Information-motivation-behavioral
CDC: Center for Disease Control and Prevention
